# Impact of Potentially Toxic Compounds in Cow Milk: How Industrial Activities Affect Animal Primary Productions

**DOI:** 10.3390/foods12081718

**Published:** 2023-04-20

**Authors:** Sergio Forcada, Mario Menéndez-Miranda, Carlos Boente, José Luis Rodríguez Gallego, José M. Costa-Fernández, Luis J. Royo, Ana Soldado

**Affiliations:** 1 Regional Service for Agrofood Research and Development (SERIDA), P.O. Box 13, 33300 Villaviciosa, Asturias, Spain; 2 Atmospheric Pollution Laboratory, CIQSO-Center for Research in Sustainable Chemistry, Associate Unit CSIC-University of Huelva, Campus El Carmen s/n, 21071 Huelva, Huelva, Spain; 3 Environmental Biogeochemistry & Raw Materials Group and INDUROT, Campus de Mieres, University of Oviedo, C/Gonzalo Gutiérrez Quirós s/n, 33600 Mieres, Asturias, Spain; jgallego@uniovi.es; 4 Department of Physical and Analytical Chemistry, Faculty of Chemistry, University of Oviedo, Avda. Julián Clavería 8, 33006 Oviedo, Asturias, Spain; jcostafe@uniovi.es; 5 Department of Functional Biology, Genetics, University of Oviedo, Avda. Julián Clavería 6, 33006 Oviedo, Asturias, Spain

**Keywords:** pollution, soil, forage, milk, polycyclic aromatic hydrocarbon, potentially toxic element

## Abstract

Potentially toxic elements (PTEs) and polycyclic aromatic hydrocarbons (PAHs) frequently coexist in soils near industrial areas and sometimes in environmental compartments directly linked to feed (forage) and food (milk) production. However, the distribution of these pollutants along the dairy farm production chain is unclear. Here, we analyzed soil, forage, and milk samples from 16 livestock farms in Spain: several PTEs and PAHs were quantified. Farms were compared in terms of whether they were close to (<5 km) or far away from (>5 km) industrial areas. The results showed that PTEs and PAHs were enriched in the soils and forages from farms close to industrial areas, but not in the milk. In the soil, the maximum concentrations of PTEs reached 141, 46.1, 3.67, 6.11, and 138 mg kg^−1^ for chromium, arsenic, cadmium, mercury, and lead, respectively, while fluoranthene (172.8 µg kg^−1^) and benzo(b)fluoranthene (177.4 µg kg^−1^) were the most abundant PAHs. Principal component analysis of the soil PTEs suggested common pollution sources for iron, arsenic, and lead. In the forage, the maximum contents of chromium, arsenic, cadmium, mercury, and lead were 32.8, 7.87, 1.31, 0.47, and 7.85 mg kg^−1^, respectively. The PAH found in the highest concentration in the feed forage was pyrene (120 µg kg^−1^). In the milk, the maximum PTE levels were much lower than in the soil or the feed forages: 74.1, 16.1, 0.12, 0.28, and 2.7 µg kg^−1^ for chromium, arsenic, cadmium, mercury, and lead, respectively. Neither of the two milk samples exceeded the 20 µg kg^−1^ limit for lead set in EU 1881/2006. Pyrene was the most abundant PAH found in the milk (39.4 µg kg^−1^), while high molecular weight PAHs were not detected. For PTEs, the results showed that soil–forage transfer factors were higher than forage–milk ratios. Our results suggest that soils and forages around farms near industries, as well as the milk produced from those farms, have generally low levels of PTE and PAH contaminants.

## 1. Introduction

Cow milk is considered a nearly complete food because of its high content of protein, fat, and essential minerals, yet the potential presence of contaminants in milk constitutes a health concern. This is particularly true in light of the fact that cow milk is one of the main constituents of the daily diet in many countries, especially for vulnerable groups, infants, and elderly people [1]. Guidelines from the European Union Common Agricultural Policy aim to ensure a high level of food safety and animal health through coherent “farm to fork” measures and adequate monitoring. This necessitates exhaustive characterization of farms at different levels of the production chain.

In the case of dairy farms, this characterization includes the milk and even the soil where crops are cultivated for animal forage (feed forage). Many dairy farms in northern Spain, for example, produce their own forage crops as a traditional practice [2], and forage-based animal nutrition depends strongly on local geographical conditions [3]. The growth of cities and industrial expansion means that many farms lie near cities or industrial zones that emit pollutants into the atmosphere and wastewater. This increases the risk of soil contamination, potentially compromising animal food safety. In fact, hazardous compounds in the soil can pose a risk to animals and to humans who consume animal-derived products [4,5]. 

Potentially toxic elements (PTEs), mainly heavy metals and metalloids, pose a growing hazard in the environment [6]. Those elements named as heavy metals are referred to by their high atomic mass and density. Their persistence in the soil, reflecting their resistance to degradation, makes them the most dangerous group of inorganic contaminants. While the PTEs iron (Fe), cobalt (Co), copper (Cu), manganese (Mn), molybdenum (Mo), and zinc (Zn) are essential for humans and cattle in trace amounts, they are toxic at higher concentrations [7,8]. The PTEs arsenic (As), mercury (Hg), cadmium (Cd), and lead (Pb) are considered toxic even at low concentrations and can cause serious illness if they accumulate in an organism [8]. PTEs can enter the food chain by first entering the soil from the atmosphere or as a result of irrigation with polluted water or deposition of animal manure, agrochemicals, and inorganic fertilizers [8,9], as well as wastewater filtration derived from industrial activities [10]. It appears that PTEs can enter forage crops and then the milk of dairy cattle that feed on that forage [9,11,12].

In addition to PTEs, polycyclic aromatic hydrocarbons (PAHs) are also widespread pollutants in soil, water, air, and plants [13,14,15]. They primarily result from the incomplete combustion or pyrolysis of organic materials, through incineration or industrial activities [4]. The U.S. Environmental Protection Agency categorizes 16 PAHs as priority pollutants due to their mutagenic and carcinogenic properties. PAHs can enter plants from the soil [16] and then transfer to the milk of dairy cows that eat the plants as forage, ultimately passing to humans who drink the milk [17,18,19]. In addition, the lipophilicity of PAHs may facilitate their accumulation in milk [20]. Considering this, the European Union has established maximum permissible limits for certain PAHs in certain foods likely to contain these contaminants. An example is milk and follow-on milk intended for infants, with a maximum permissible content of 1 µg L^−1^ of benzo(a)pyrene or 1 µg L^−1^ of the sum of benzo(a)pyrene, benzo(a)anthracene, benzo(b)fluoranthene, and chrysene [21]. 

PTEs and PAHs frequently coexist in soils in proximity to highways and certain industries, such as the smelting and mining industries [22,23]. Therefore, it is quite important to assess the risk that milk produced from dairy farms near these areas may be contaminated with PAHs and PTEs. Northern Spain provides a good study area, since many dairy farms are located close to active industrial facilities. Here, we quantified several PTEs and PAHs of concern in soil, forage, and milk samples on farms near and farther from industrial areas in northern Spain, and we evaluated the transfer of these contaminants into milk.

## 2. Materials and Methods

### 2.1. Reagents

Ultrapure water (≥18 MΩ-cm resistivity; ≤5 µg L^−1^ TOC) was obtained from a Milli-Q IQ 7000 purification system (Merck Millipore, Darmstadt, Germany). Elemental calibration solutions for inductively coupled plasma mass spectrometry (ICP-MS) were obtained from HPS (North Charleston, Charleston, SC, USA) and were prepared as 1 g L^−1^ solutions in 1% nitric acid. All dilutions were performed with analytical-grade 65% nitric acid (Suprapur^®^) or 30% hydrochloric acid (Merck, Darmstadt, Germany). Our analytical procedures were validated using the following Certified European Reference Materials (IRMM, Geel, Belgium): “ERM-CC141 Loam soil”, “ERM-CD281 Rye grass”, and “ERM-BD151 Skimmed milk powder”.

### 2.2. Sample Collection 

**Figure 1:**
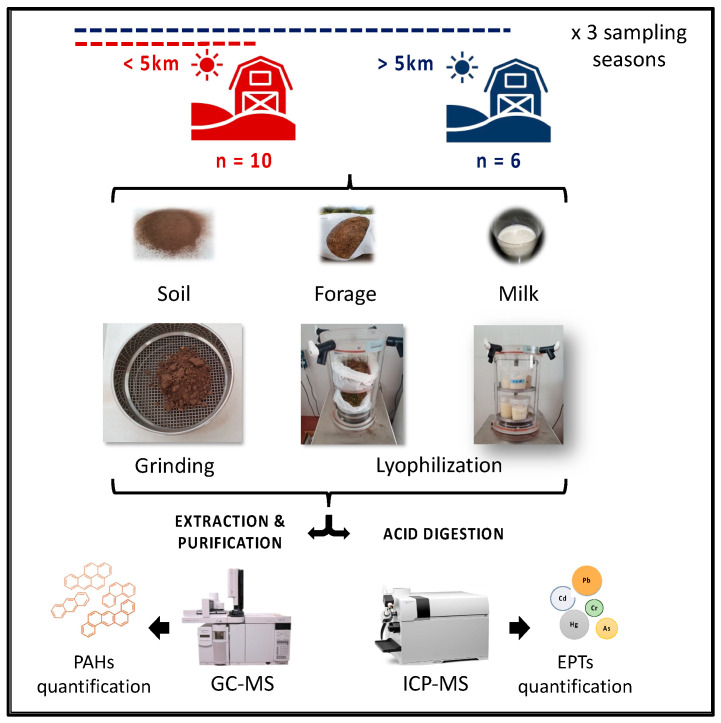
Sampling collection and processing of soil, forage, and tank milk from the selected farms.

Soil, milk, and forages (fresh or silage, depending on the farm stock) were sampled at 16 dairy farms, each of which had no more than 40 heads of cattle. As the studied area comprises multiple pollution sources, 10 farms were classified as close to industries (having one or more pollution sources less than 5 km away), while 6 farms were grouped as far (located 5 km or more from each pollution source). This 5 km distance was chosen to provide a compromise between the total farms sampled—those at maximum distance (classified as far) and those near to industries. Sampling on each farm was performed in autumn, spring, and summer in order to assess reproducibility. The soil samples from the upper layer soil (20 cm) were collected in three random points per cropland with a Dutch auger of 5 cm inner diameter. Once in the laboratory, the samples were air-dried at room temperature, crumbled, finely crushed, and sieved through a 2 mm screen [9,11,24]. Forage samples consisted of grass-based fodder and they were either fresh or preserved as silage (grass chopped and packaged without air to facilitate the fermentation process and minimize nutrient losses, for use as animal feed). Forage samples were collected at three points in the trough, then pooled to a total mass of around 1 kg. Milk samples (1 L) were collected directly from the tank after stirring. Figure 1 summarizes the sample collection and processing.

### 2.3. Sample Preparation 

Soil samples were dried, ground, and sieved through a 2 mm sieve. Freshly collected samples of forage, silage, and total mixed rations were freeze-dried in a Coolsafe Pro 100-9 system (Labogene, Allerød, Denmark), ground, and stored at room temperature until analysis. Milk was freeze-dried under the same conditions and stored at −80 °C until analysis (see Figure 1). Samples were analyzed within a maximum time of six months.

### 2.4. Determination of Inorganic Elements in Soil, Forage, and Milk by ICP-MS

Procedures to quantify inorganic elements were based on official methods for trace element determination by ICP-MS: ISO/TS 16965:2013, EN 17053:2018 and ISO 15151:2018 for soils, animal feed, and milk, respectively. All of them were self-optimized and validated in our laboratory by using different European reference materials (Joint Research Centre, EU): loam soil (ERM CD-141), rye grass (ERM CD-281), and skimmed milk powder (ERM BD-151). Accuracy, reproducibility, and optimization of these procedures are detailed in Appendix A.

Samples of soil (0.1 g), forage (0.5 g), or milk powder (0.5 g) were digested in 8 mL of aqua regia (HCl:HNO_3_ 3:1) in closed polytetrafluoroethylene vessels using an Ethos One microwave digestion system (Milestone Srl., Sorisole, BG, Italy), as described in Table A2. The digested solution was filtered through a 0.22 µm syringe filter (Merck Millipore, Billerica, MA, USA), then diluted to 40 mL with ultrapure water in the case of forage and milk samples [overall dilution, 1:80 (*w*/*v*)] or 20 mL in the case of soil samples. An aliquot (1 mL) of diluted soil samples was then diluted to 10 mL [overall dilution, 1:2000 (*w*/*v*)]. 

Standard metal solutions were prepared daily. Solutions of Na, K, Mg, and Ca were prepared from a multi-elemental stock solution (1000 µg mL^−1^), while solutions of Cr, Zn, Fe, Cu, As, Se, Cd, and Pb were added from individual stock solutions (1000 µg mL^−1^). All standards were prepared in 1% HNO_3_. Internal standards (HPS, North Charleston, Charleston, SC, USA) were as follows: ^45^Sc for Na, K, Ca, and Mg; ^72^Ge for Cr, Fe, Cu, Zn, Se, and As; ^103^Rh for Cd; and ^193^Ir for Hg and Pb. Samples were analyzed per duplicate.

The content of inorganic elements in the soil and forage samples was calculated in terms of dry weight, while the content of inorganic elements in the milk was calculated in terms of wet weight after applying a correction factor based on mean water content (88%) [25]. The assay procedures were validated per triplicate for the three sample matrices using the Certified European Reference Materials, as set out in Section 2.1. Elements whose concentrations were not reported in the reference materials were spiked into the materials. Recoveries in ryegrass ranged from 93% for Cd to 114% for Ca, while those in skimmed milk powder ranged from 88% for Pb to 113% for Cr (Table A2).

### 2.5. Determination of PAHs in Soil, Forage, and Milk by Gas Chromatography–Tandem Mass Spectrometry (GC-MS/MS)

Soil samples (10 g) were extracted with dichloromethane:acetone [1:1 (*v*/*v*)] in a Soxtherm system (Gerhardt, Bonn, Germany). The extracts were cleaned with silica gel, then concentrated by rotary evaporation (Heidolph, Schwabach, Germany). PAH concentrations were determined after injection into a 7890A GC System coupled to a 5975C Inert XL MSD with a Triple-Axis Detector (Agilent Tech., Santa Clara, CA, USA), following EPA Method 8272, with modifications. The samples were run on a capillary column DB-5ms with a length of 30 m, an inner diameter of 0.25 mm, and a film thickness of 0.25 μm (Agilent Tech. Santa Clara, CA, USA), with He as the carrier gas at 1 mL min^−1^. The initial oven temperature of 70 °C was held for 2 min; ramped up to 220 °C at 20 °C min^−1^, then to 270 °C at 10 °C min^−1^, where it was held for 1 min; ramped up to 290 °C at 10 °C min^−1^, where it was held for 1 min; and finally ramped up to 300 °C at 10 °C min^−1^, where it was held for 7 min. The total run time of GC separation was 30 min The gas chromatography injector was operated in splitless mode for 2 min, and its temperature was maintained at 260 °C. The mass spectrometer was operated in electron ionization mode (EI) at 70 eV and calibrated daily by auto-tuning with perfluorotributylamine (PFTBA). PAH calibration standards (AccuStandard, New Haven, CT, USA) were used. Blanks (one for every five samples), duplicate samples, and cross correlation were used for quality assurance and quality control (QA/QC) purposes. RSD for individual PAHs was below 10% in all cases. The following species (*m*/*z*) were quantified: 128 (naphthalene), 152 (acenaphthylene), 153 and 154 (acenapthene), 165 and 166 (fluorene), 178 (anthracene/phenanthrene), 202 (fluoranthene/pyrene), 228 (benzo(a)anthracene/chrysene), 252 (benzo(b)fluoranthene/benzo(k)fluoranthene), 276 (indene(1,2,3-c,d)pyrene/benzo(g,h,i)perylene), and 278 (benzo(a,h)anthracene). In Appendix A, the most representative chromatogram of soil samples has been included.

Forage and milk samples were treated according to the “QuEChERS” extraction method, with some modifications [26]. Briefly, samples (10 g) were extracted with 30 mL acetonitrile and vortexed at 3000 rpm for 1 min. A total of 4 g anhydrous MgSO_4_ and 1 g NaCl were added and immediately vortexed for 1 min, then 50 µL of an internal standard solution were added and the mixture was vortexed for another 30 s. The mixture was centrifuged at 2800× *g* for 5 min at room temperature and the supernatant was purified by a dispersive solid-phase extraction method [26]. An aliquot of supernatant (5 mL) was transferred to a flat-bottomed flask, concentrated in a 40 °C water bath until near-drying, and dissolved in 5 mL of cyclohexane [26]. An aliquot of 1 µL was injected into a 7890B gas chromatograph (Agilent Tech.) equipped with a Select PAH CP7462 capillary column with a length of 30 m, an inner diameter of 0.25 mm, and a film thickness of 0.15 µm. He was used as carrier gas at 2 mL min^−1^. The initial oven temperature of 70 °C was held for 0.7 min, ramped up to 180 °C at 85 °C min^−1^ and then to 230 °C at 3 °C min^−1^, where it was held for 7 min; ramped up to 280 °C at 28 °C min^−1^, where it was held for 10 min; and, finally, ramped up to 350 °C at 14 °C min^−1^, where it was held for 3 min. The total run time of GC separation was 60 min. The GC injector was operated in splitless mode for 1 min and its temperature was maintained at 300 °C. The compounds were detected using a 7000D mass spectrometer (Agilent Tech.), which was operated in electron ionization mode (EI) at 70 eV. The following *m*/*z* ratios were monitored: 178 (anthracene/phenanthrene), 202 (fluoranthene/pyrene), 228 (benzo(a)anthracene/chrysene), 252 (benzo(b)fluoranthene/benzo(k)fluoranthene/benzo(a)pyrene), 276 (benzo(g,h,i)perylene/Indene(1,2,3-c,d)pyrene), and 278 (benzo(a,h)anthracene). Each sample was analyzed per duplicate. The method was validated using five internal standards (AccuStandard, New Haven, CT, USA), prepared by adding isotopically labelled PAHs to sample extracts. Concentrations of PAHs in the samples were determined by comparing their peak areas to those of the internal standards.

### 2.6. Data Treatment and Statistical Analyses

Univariate statistical descriptors (mean, median, coefficient of variation, minimum, and maximum) were calculated for the concentrations of PTEs and PAHs in each type of sample. The variation (%) in the mean concentration for each metal or PAH between the close and far groups of farms was calculated using the following expression:

(1) $$Variation\left(\%\right)=\frac{{mean}_{close}}{{mean}_{far}}\cdot 100$$

Principal component analysis was performed for soil data to identify anthropogenic or natural factors associated with the concentrations of contaminants. Factors were extracted using the Kaiser/Gutmann criterion and varimax rotation, reflecting recommendations and our own experience [27,28,29].

The soil–forage transfer factor (TF_sf_) and forage–milk transfer factor (TF_fm_) for the inorganic elements were calculated as follows:

(2) TFsf=CfCs

(3) TFfm=CmCf

where C_f_, C_s_, and C_m_ are the median concentrations in the forage, soil and milk, respectively. SPSS 24 (IBM, Armonk, NY, USA) was used for all statistical analyses. 

## 3. Results and Discussion

### 3.1. PTEs and PAHs in the Soil 

The results of the soil analyses are summarized in Table 1. Descriptive statistics are detailed for each element (including PTEs and essential minerals) and PAHs analyzed. The soils closer (<5 km) to industrial areas contained higher content of PTEs and heavy weight PAHs than those located farther away (>5 km).

The similitude between the mean and the median is a preliminary indicator of normal distribution. The variation (V%) revealed an enrichment of PTEs and PAHs in soils closer to industrial areas (Table 1), with the highest value for dibenzo(a,h)anthracene (89%). The enrichment of Zn, Cd, and Pb was consistent with the known metal emissions from current and past industrial activities in this region of northern Spain [30,31]. PAHs with a molecular weight higher than that of fluoranthene were also enriched in the soil closer to industrial areas, except for pyrene (38%), and these results are consistent with studies of soils near industrial areas in northern Spain [32,33]. The enrichment of these high molecular weight PAHs is concerning, as these are the most persistent PAHs in the environment. In addition, these data are consistent with previous studies on soils located near to the industrial areas [32,33,34,35].

To assess the risk that the observed levels of pollutants may pose for humans and the environment, we compared the measured levels to so-called “risk-based soil screening levels” (RBSSLs) [36], which are based on toxicity parameters for different uses of soil (Table 1). We applied the most restrictive values for “other uses” of soil, which include farming [36]. 

In the soils close to industries, Cu, Zn, As, Cd, Hg, and Pb exceeded the threshold limits by at least 100%. For instance, the mean concentration of Hg (0.97 mg kg^−1^), one of the most toxic elements, was close to its RBSSL (1 mg kg^−1^). In the case of soils located more than 5 km away from industrial areas, thresholds were occasionally exceeded only for Cu and Hg, and mean values were much lower than RBSSLs. In the case of PAHs, the concentration of benzo(a)pyrene in the soils closer to industries (45.4 µg kg^−1^) was more than twice the RBSSL (20 µg kg^−1^), while it was notably lower in the soils farther away (16.7 µg kg^−1^). More specifically, the soils from N1, N2, and N3 dairy farms showed levels of benzo(a)pyrene above their ML, with 85.9, 61.4, and 78.7 ug kg^−1^, respectively. These three farms are located less than 2 km from the steel industry and less than 5 km from the zinc industry. Similar enrichment in heavy-molecular-weighted PAHs has been previously reported in soils located less than 2 km from a Cu smelting industry [37]. These results suggest that livestock near industrial areas may be exposed to above-threshold levels of several pollutants when they feed on forage cultivated on local soils.

To identify potential pollution sources, principal component analysis was performed using all the samples, irrespective of their location (Table 2). Four principal components explained 83% of the initial variance with high communality values. PTEs such as As and Pb were quite well represented by principal component 1, which was also associated with high Fe and Se load, suggesting the presence of an anthropogenic source that was probably related to the steel industry (Fe) and/or coal-combustion (Fe and Se) power plants [27,38]. This component 1 was also associated with natural iron oxy-hydroxides, which may explain the presence of As. The elements with higher loads in the second principal component were Mg, Ca, and K, which were probably associated with natural sources, such as calcareous and clayey materials. In the third principal component, a remarkable association was observed among high concentrations of PAHs, Zn, and Cd, consistent with emissions from the Zn smelting industry [32]. The correlation between Zn and heavy molecular-weighted PAHs has been also observed in soils near Cu smelting industries [37]. The high levels of PAHs and the contribution of Pb in the third component, together with the absence of PAHs in the other two components, may indicate heavy-traffic pollution as another source [39]. The fourth principal component was linked to Na and Cr, both naturally occurring elements.

### 3.2. PTEs and PAHs in Forage

Table 3 shows the concentration (mean, median, minimum, and maximum) and the percentage of variation between mean (and median) concentration of inorganic elements and PAHs in the forages produced near to (<5 km) and farther from (>5 km) the point-sources of pollution. The concentrations of PTEs and PAHs in the forage were generally lower than those measured in the soils, suggesting limited transfer from soils to plants [27]. This could be explained by the low bioavailability of PTEs in the soils of the industrial areas [27], and perhaps by low deposition from the atmosphere. 

The number of pollutants enriched closer to the industry was smaller in the forage than in the soil (Table 1 and Table 3), although in both types of samples, Zn, Cd, and PAHs with at least four aromatic rings were enriched closer to industrial areas. This enrichment in high-molecular-weight PAHs in the soils and plants can be partially explained by the “distillation effect” [40]: high molecular weight PAHs in the atmosphere deposit onto surfaces closer to their source, whereas low-molecular weight PAHs diffuse farther before deposition. The levels of PAHs found in forage samples (1–20 µg kg^−1^ dry weight) were lower than those reported in 2003 in grasslands near roads with high-traffic intensity [20], but they were similar to those in forages from urban and rural farms [19].

Forage (fresh forage or silage) is the primary source of essential mineral supply to cattle in sustainable farms [7]. The essential trace minerals are required in the diet of the animals, as they play fundamental roles in their organisms, such as the roles of enzyme cofactors, catalyzers of metabolic reactions, and so on [7]; however, they become potentially toxic at high concentrations, so the National Research Council (NRC, United States) has established tolerable limits for these elements in the cattle diet. Nearly all the essential trace minerals (Zn, Cu, Se, and Cr) were below the maximum tolerable limits that the NRC recommends for cattle [41]. The only exception was Fe, whose median concentration (920 mg kg^−1^ dry weight) in farms near industries exceeded the tolerable level of 500 mg kg^−1^, which was much higher than the concentration found in farms far away from industrial areas.

Among the PTEs, Cd and Pb showed respective median concentrations of 0.115 and 1.10 mg kg^−1^ in the forage, which were below the levels in forage produced near industrial activities in Romania [11] or India [42] but above the levels on commercial farms in England [43]. The maximal content of As (7.87 mg kg^−1^), Cd (1.31 mg kg^−1^), and Hg (0.47 mg kg^−1^) in the forage exceeded the maximum levels (ML) for animal feed based on European Union regulations [44,45]. More specifically, one farm exceeded the ML of Cd (N1, see Table A1) with 1.31 mg kg^−1^. In that sense, other farms near industries (N2 and N3) also had high levels of Cd (>0.6 mg kg^−1^), although they did not exceed its ML. In contrast, the As concentration in the forages was above its ML in farm F4 (7.87 mg kg^−1^), which is more than 20 km away from point pollution sources (Table A1), and in the close-to-industry farms N1 (3.94 mg kg^−1^) and N4 (2.19 mg kg^−1^). Again, the Hg concentration exceed its ML in farm F4 (0.47 mg kg^−1^) and in the close-to-industry farms N4 (1.13 mg kg^−1^) and N7 (1.14 mg kg^−1^). Thus, As and Hg might not be enriched near industrial facilities, which is similar to what we observed in the soil. These results suggest that As and Hg, in particular, may have a natural occurrence. 

Regarding PAHs, EU legislation has not established a ML in animal feed for these compounds. However, the most concerning PAH is benzo(a)pyrene, whose concentrations were higher in the forages produced in farm N2 (48.7 µg kg^−1^), which is located 0.4 km from the steel industry (Table A1). Together with benzo(a)pyrene, the forage from this farm also contained higher concentrations of the rest of PAHs.

### 3.3. PTEs and PAHs in Milk

Table 4 provides a comparison between the concentrations of inorganic elements and PAH found in the milk produced in farms close to and farther from industries. In addition, similarly to the soil and the forage, the variation in the mean (and median) concentration between both locations was calculated for each pollutant to assess its enrichment in the milk, depending on industrial proximity.

The concentrations of PTEs in the milk were low, regardless of whether the farms were near to or farther from industrial areas. Hg and Pb showed substantial enrichment (50%) in farms closer to industries, while Cr, As, and Cd showed weaker enrichment (35%). These results are consistent with previous studies showing that the milk of cows on farms near industrial areas contained elevated contents of Cd [46] and Pb [47]. Nevertheless, the levels of Cd and Pb in milk were considerably higher in those studies than in the present work. Indeed, the levels of Cd in 11% of our milk samples and the level of Hg in 63% of our milk samples were below the limit of detection of our methodology (see Table 4). None of the milk samples exceeded the maximum recommended limit of 20 µg kg^−1^ for Pb [48] (The European Union has not established limits in milk or dairy products for the other metals that we analyzed). None of our samples exceeded the maximum level of 2.6 µg kg^−1^ for Cd, as recommended by the International Dairy Federation [49]. Perhaps these PTEs could be accumulated in the liver, kidney, or lung bovine organs, as previously stated [50].

The presence of PAHs in the milk was addressed to a lesser extent than the presence of PTEs; however, some works have reported PAH concentrations in milk from cows raised in industrial or in rural areas [4,18,19]. The presence of PAHs in milk can occur not only after ingestion of soil when livestock graze in fields, but also via feed (pasture or silage) when livestock is confined indoors [4].

We detected only three PAHs in the milk, and all three had low molecular weight: phenanthrene, fluoranthene, and pyrene. Their concentrations were similar to those reported in rural areas of France [18,19]. In contrast to the enrichment that we observed in the soil and the forage, we did not observe such enrichment in the milk, which was similar to a report comparing PAH levels in milk from rural or urban areas in France [19]. These results suggest that PAHs are not efficiently transferred into milk at such levels of pollution, as previously reported in a controlled experiment with goats, where C^14^ PAHs were added to the diet [51]. 

### 3.4. Transfer Factors between Soil and Forage and between Forage and Milk

Table 5 provides the soil–forage transfer factor (TF_sf_) and forage–milk transfer factor (TF_fm_) for the inorganic elements.

In general, TF_sf_ ratios were higher than TF_fm_ ratios. Previous studies also reported very low forage–milk transfer of heavy metals, with values as low as 1:500 [52], implying that mammary glands act as barriers to prevent the entry of PTEs [53]. Na, K, Ca, and Mg had higher TF_sf_ and TF_fm_, probably reflecting that they are major essential elements. TF_sf_ values were above 1 for these elements, indicating a higher concentration of these minerals in the forage than in the soil. TFs varied across studies (Table 5), probably reflecting the complex influences on these factors, including plant species, soil properties, and dry matter intake by the animals [11]. Moreover, these TF_sf_ could be affected by the sampling procedure, so it is necessary to remark that these data were obtained by collecting three subsamples in each location.

**Table 5:** Soil–forage (TF_sf_) and forage–milk (TF_fm_) transfer factors for potentially toxic metals.

	TF_sf_				TF_fm_			
	Present Study			Literature	Present Study			Literature
	Mean ± SD	Range	Median		Mean ± SD	Range	Median	
Na	20.4 ± 50.9	0.65–401	12.80		0.227 ± 0.329	0.047–1.973	0.1212	
K	3.0 ± 2.4	0.62–11.5	2.517		0.118 ± 0.054	0.042–0.345	0.1185	
Ca	2.2 ± 1.8	0.24–7.14	1.626		0.157 ± 0.058	0.076–0.41	0.1538	
Mg	1.1 ± 0.8	0.1–3.77	1.062		0.051 ± 0.019	0.032–0.107	0.0483	
Cr	0.47 ± 0.76	0.023–3.95	0.232	(0.01–0.1) [54]	0.002 ± 0.007	0–0.046	0.0004	
Fe	0.005 ± 0.006	0.00–0.033	0.004		0.0005 ± 0.0005	0.00003–0.0017	0.0002	
Cu	0.74 ± 0.53	0.082–2.02	0.682	0.07 [11] (0.1–1) [54]	0.0048 ± 0.0024	0.0014–0.0125	0.0048	0.04 [11]
Zn	0.54 ± 0.37	0.048–1.85	0.460	0.092 [11] (1–10) [54]	0.074 ± 0.045	0.016–0.173	0.0636	0.117 [11]
As	0.099 ± 0.28	0.003–1.84	0.033	(0.01–0.1) [54]	0.0074 ± 0.0077	0.0002–0.038	0.0050	
Se	0.187 ± 0.146	0.026–0.602	0.141	(0.1–10) [54]	0.0010 ± 0.001	0–0.005	0.0007	0.037 [11]
Cd	0.338 ± 0.407	0.011–2.31	0.235	0.06 [11] (1–10) [54]	0.0002 ± 00003	0–0.0013	0.0001	0.021 [11]
Hg	0.125 ± 0.202	0.002–0.48	0.041	(0.01–0.1) [54]	0.001 ± 0.005	0–0.029	0.0000	
Pb	0.062 ± 0.087	0.002–1.0799	0.029	0.005 [11] 0.01–0.1 [54]	0.0010 ± 0.001	0–0.005	0.0007	0.037 [11]

SD: standard deviation.

The TF_sf_ values in our work followed the trend Zn ≈Cu > Cd >> Pb, showing some discrepancies with previous work in the transfer of Cu and Zn [11,54], but consistent with a report that Zn and Cu accumulate to a much greater extent than Cd in edible plant parts [55]. Our trend is also consistent with the lower transfer of Pb from soil to plants observed in previous work, which led investigators to propose that this metal enters the human food chain via an alternative water–forage–milk pathway [9]. The TF_fm_ values in our work followed the trend Zn > Cu > Pb > Cd, consistent with previous studies in Romania [11]. In contrast to that work, however, PTE concentrations in the present study were orders of magnitude larger in the forage than in the milk.

## 4. Conclusions

Our results suggest that PTEs and high-molecular-weight PAHs are enriched in soils near industrial areas, and that this enrichment led to somewhat elevated levels in the forage but not dangerously high levels in the milk (lower than the EU legislation maximum permitted level) from cows feeding on that forage. These results suggest that there is no risk for humans consuming cow’s milk from these areas. Principal component analysis suggested that the sources of soil pollutants may be related to anthropogenic factors linked to industrial activity, as well as to natural soil mineralogy, as found in principal component 1 for As, Pb, Fe, and Se, emitted because of coal combustion of power plants or the steel industry. The calculated forage–milk transfer factors proved to be minimal for the most toxic elements (Cd, Hg, and Pb), with values lower than 10^−3^. Further, the content of PAHs and PTEs decreased along the soil–forage–milk food chain and only low molecular weight PAHs were detected in the milk. Future work should examine the fate of PTEs and PAHs in soils and farm-produced forage, as well as meat production and the health implications for cattle.

## Figures and Tables

**Table 1:** Comparison of metals and polycyclic aromatic hydrocarbons in soils from farms located <5 or >5 km from industrial areas.

	Farms < 5 km from Industrial Areas (*n* = 10)				Farms > 5 km from Industrial Areas (*n* = 6)				Total Median	V (%)	RBSSL
	Mean	Median	Min	Max	Mean	Median	Min	Max			
Inorganic elements											
Na (g kg^−1^)	3.10	2.81	0.08	9.55	3.24	2.95	0.35	14.6	2.94	−5	-
Mg (%)	0.44	0.22	0.08	1.34	0.32	0.17	0.1	0.79	0.20	27	-
K (%)	0.77	0.64	0.24	2.11	0.63	0.53	0.34	1.7	0.59	19	-
Ca (%)	0.73	0.55	0.1	2.34	0.62	0.35	0.14	2.3	0.43	15	-
Cr (mg kg^−1^)	35.1	24.4	2.7	141.5	24.8	22.2	8.0	56.0	23.3	29	-
Fe (%)	2.58	1.92	0.85	6.09	1.54	1.45	0.9	2.32	1.76	40	-
Cu (mg kg^−1^)	20.8	19.9	4.6	56.0	17.0	11.0	7.1	71.8	15.4	18	55
Zn (mg kg^−1^)	261	232	30	506	96	61	33	256	176	63	455
As (mg kg^−1^)	17.8	11.3	4.9	46.1	10.3	10.4	4.3	14.5	11.1	42	40
Se (mg kg^−1^)	1.75	1.59	1.1	3.09	1.42	1.43	0.95	1.86	1.50	19	25
Cd (mg kg^−1^)	1.41	1.16	0.21	3.67	0.39	0.32	0.18	1.24	0.71	73	2
Hg (mg kg^−1^)	0.97	0.41	0.06	6.11	0.57	0.19	0.06	3.2	0.39	41	1
Pb (mg kg^−1^)	52	48	13	138	25	24	15	43	34	51	70
Polycyclic aromatic hydrocarbons (µg kg^−1^)											
Naphthalene	2.75	<0.1	<0.1	13.86	1.45	<0.1	<0.1	5.61	3.73	47	1000
Acenaphthylene	1.54	0.98	<0.1	7.69	1.23	0.71	<0.1	5.23	1.02	20	-
Acenaphthene	0.63	<0.1	<0.1	7.81	0.81	0.55	<0.1	2.59	1.19	−28	6000
Fluorene	2.36	2.03	<0.1	9.61	1.06	1.13	<0.1	2.47	1.41	55	5000
Anthracene	28.8	25.9	0.67	85.2	17.5	12.8	2.78	49.19	20.83	39	45,000
Phenanthrene	4.86	3.33	<0.1	22.95	3.59	1.12	<0.1	17.11	2.76	26	-
Fluoranthene	57.9	45.7	2.7	172.8	28.9	18.8	4.4	109.5	34.6	50	8000
Pyrene	38.5	30.5	1.4	100.6	24.0	15.1	3.7	94.6	25.7	38	6000
Benzo(a) anthracene	36.7	26.0	3.7	95.4	15.5	9.1	3.2	58.3	19.7	58	200
Crysene	48.2	35.5	4.1	116.4	18.6	10.3	3.1	55.7	27.4	61	20,000
Benzo(b) fluoranthene	83.2	61.7	8.0	177.4	35.0	22.2	7.0	108.8	52.4	58	200
Benzo(k) fluoranthene	25.2	17.5	2.3	56.1	9.5	4.8	1.5	27.5	14.2	62	2000
Benzo(a)pyrene	45.4	30.3	3.8	105.3	16.7	8.8	2.9	50.0	22.3	63	20
Indene(1,2,3-c,d)pyrene	30.5	26.6	<0.1	81.9	7.4	<0.1	<0.1	38.8	32.4	76	300
Dibenz(a,h) anthracene	10.4	6.1	<0.1	47.5	1.2	<0.1	<0.1	9.4	10.7	89	30
Benzo(g,h,i) perylene	39.7	29.8	6.5	91.7	13.2	8.6	3.6	42.9	21.3	67	-

Max: maximum; min: minimum; *n*: number of farms; V: close-far variation; <0.1: not detected; RBSSL: risk-based soil-screening level.

**Table 2:** Principal component data matrix (rotated) for potentially toxic elements and polycyclic aromatic hydrocarbons (PAHs) in soils.

Element	Principal Component				Communality
	1	2	3	4	
As	0.922	0.125	0.158	0.019	0.891
Se	0.862	0.242	0.125	0.126	0.833
Fe	0.781	0.432	0.257	−0.006	0.862
Pb	0.719	−0.020	0.626	0.050	0.913
Mg	0.211	0.944	−0.017	0.059	0.939
Ca	−0.006	0.926	0.190	0.151	0.917
K	0.398	0.788	−0.101	0.357	0.917
Cu	0.306	0.727	0.489	0.099	0.872
Zn	0.320	0.265	0.838	0.136	0.893
Sum PAHs	−0.035	0.044	0.807	−0.154	0.678
Cd	0.449	−0.063	0.791	0.171	0.861
Hg	0.093	0.207	0.539	0.464	0.557
Na	−0.095	0.138	0.029	0.887	0.816
Cr	0.460	0.180	0.032	0.616	0.625
% VE	45.840	63.970	73.852	82.653	

VE: Variance explained (cumulative).

**Table 3:** Comparison of potentially toxic metals and polycyclic aromatic hydrocarbons in feed from farms <5 or >5 km from industrial areas.

	Farms < 5 km from Industrial Areas (*n* = 10)				Farms > 5 km from Industrial Areas (*n* = 6)				Total Median	V (%)	ML
	Mean	Median	Min	Max	Mean	Median	Min	Max			
Inorganic elements											
Na (g kg^−1^)	3.61	3.9	0.44	6.48	2.71	2.92	0.2	5.68	3.16	25	-
Mg (g kg^−1^)	2.32	2.34	1.2	3.15	2.28	1.94	1.32	4.15	2.30	2	-
K (g kg^−1^)	17.5	15.13	4.91	40.95	15.56	13.2	10.07	29.59	13.75	11	-
Ca (g kg^−1^)	7.72	7.63	3.05	10.52	8.23	7.74	5.42	16.05	7.63	−7	-
Cr (mg kg^−1^)	7.56	6.51	1.57	19.12	8.04	5.58	0.96	32.76	6.07	−6	-
Fe (g kg^−1^)	1.28	0.92	0.24	7.63	0.68	0.48	0.12	2.55	0.82	47	-
Cu (mg kg^−1^)	8.86	7.41	4.44	18.96	10.58	9.4	5.12	22.8	7.48	−19	-
Zn (mg kg^−1^)	89.8	76.3	22.9	216.1	43.9	36.2	24.7	87.2	61.7	51	-
As (mg kg^−1^)	0.8	0.53	0.15	3.94	0.85	0.3	0.06	7.87	0.42	−7	2 ^a^
Se (µg kg^−1^)	300	209	50	1077	287	242	38	729	220	4	-
Cd (µg kg^−1^)	318	192	39	1313	94	87	10	314	115	70	1000 ^b^
Hg (µg kg^−1^)	38.9	27.3	11.4	114.3	39.2	7.9	3.0	471.1	20.1	−1	100 ^a^
Pb (mg kg^−1^)	1.83	1.5	0.23	7.85	1.48	0.87	0.2	5.69	1.10	19	10 ^a^
Polycyclic aromatic hydrocarbons (µg kg^−1^)											
Phenanthrene	7.68	7.4	3.3	14.7	6.69	6.9	3.5	10.4	7.1	13	-
Fluoranthene	15.2	14.3	4.5	37.4	10.4	7.1	1.8	26.1	10.6	32	-
Pyrene	34.0	23.9	3.4	120	23.5	15.7	1.1	75.3	19.6	31	-
Benzo(a)anthracene	3.17	1.9	<0.1	31.3	0.05	<0.1	<0.1	0.9	1.9	98	-
Crysene	5.8	3.45	1.2	51.9	1.11	1.2	<0.1	2.5	2.1	81	-
Benzo(b)fluoranthene	6.91	3.2	1.5	71.3	0.68	<0.1	<0.1	3.2	2.1	90	-
Benzo(k)fluoranthene	2.8	1.5	<0.1	30	0.07	<0.1	<0.1	1.2	1.7	98	-
Benzo(a)pyrene	4.28	2.15	<0.1	48.7	0.06	<0.1	<0.1	1	2.3	99	-
Indene(1,2,3-c,d)pyrene	3.92	1.9	0.9	41.6	0.24	<0.1	<0.1	1.5	1.6	94	-
Dibenz(a,h)anthracene	0.68	<0.1	<0.1	10.2	<0.1	<0.1	<0.1	<0.1	1.4	100	-
Benzo(g,h,i)perylene	4.76	2.25	1	54.9	0.19	<0.1	<0.1	1.5	1.9	96	-

Max: maximum; min: minimum; *n*: number of farms; V: close–far variation; <0.1: not detected; ML: maximum level according to EU regulations; ^a^ (EU 2019/1869); ^b^ (EU 1275/2013).

**Table 4:** Comparison of metals and polycyclic aromatic hydrocarbons in milk from farms < 5 or > 5 km from industrial areas.

	Farms < 5 km from Industrial Areas (*n* = 10)				Farms > 5 km from Industrial Areas (*n* = 6)				Total Median	V (%)	ML
	Mean	Median	Min	Max	Mean	Median	Min	Max			
Inorganic elements											
Na (g kg^−1^)	0.39	0.39	0.29	0.52	0.41	0.41	0.34	0.50	0.40	−5	-
Mg (g kg^−1^)	0.11	0.11	0.08	0.13	0.12	0.11	0.1	0.15	0.11	−8	-
K (g kg^−1^)	1.66	1.69	1.37	1.89	1.71	1.72	1.51	1.97	1.70	−3	-
Ca (g kg^−1^)	1.15	1.15	1	1.27	1.23	1.21	1.16	1.53	1.19	−7	-
Cr (µg kg^−1^)	8.56	3.09	<0.07	74.1	5.14	1.09	<0.07	44.2	1.88	40	-
Fe (mg kg^−1^)	0.27	0.18	0.05	1.22	0.25	0.19	0.08	1.19	0.18	9	-
Cu (µg kg^−1^)	36.6	36.0	19.6	58.6	42.9	43.4	26.5	73.6	40.2	−17	-
Zn (mg kg^−1^)	3.52	3.46	2.92	4.24	4.04	4.12	3.02	5.57	3.67	−15	-
As (µg kg^−1^)	2.77	1.62	1.23	16.01	1.83	1.7	1.26	3.91	1.65	34	-
Se (µg kg^−1^)	28.7	25.8	17.7	54.0	28.6	27.3	12.6	55.5	27.2	1	-
Cd (µg kg^−1^)	0.03	0.02	<0.002	0.12	0.02	0.02	<0.002	0.03	0.019	38	-
Hg (µg kg^−1^)	0.03	<0.01	<0.01	0.28	0.01	<0.01	<0.01	0.17	<0.01	59	-
Pb (µg kg^−1^)	1.17	0.95	0.2	2.7	0.54	0.51	<0.06	1.44	0.70	54	20 ^a^
Polycyclic aromatic hydrocarbons (µg kg^−1^)											
Phenanthrene	1.18	1.11	0.16	3.04	1.1	0.89	0.41	3.55	1.07	7	-
Fluoranthene	1.91	1.1	0.22	7.22	1.44	0.92	0.32	6.29	1.03	25	-
Pyrene	7.73	3.49	0.43	39.4	5.8	3.6	0.64	20.0	3.55	25	-

Max: maximum; min: minimum; V: close-far variation; ML: Maximum Level according to EU regulations; *n*: number of farms. ^a^: (CE 1881/2006).

**Figure A1:**
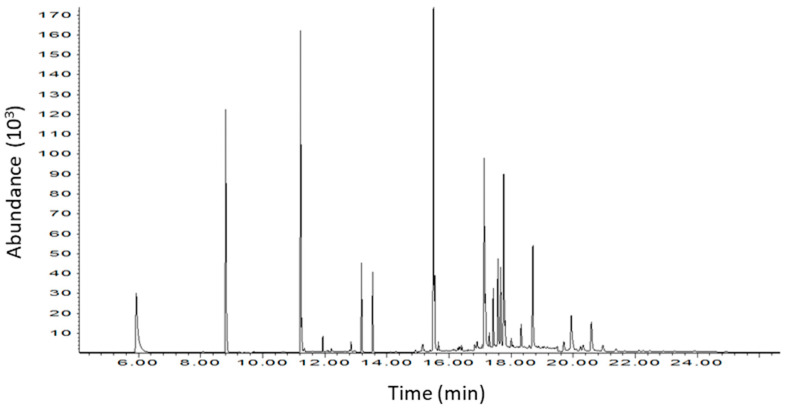
Merged-SIM chromatogram of PAHs found in a representative sample of soil collected in the vicinity of the industrial areas. Concentrations found in μg·kg^−1^ (main peaks): naphthalene (1.04), acenaphthylene (0.71), acenaphtene (0.64), Fluorene (0.61), phenanthrene (9.73), anthracene (0.89), fluoroanthene (10.23), pyrene (7.98), benzo(a)anthracene (5.06), chrysene (7.06), benzo(k)fluoroanthene (14.68), benzo(b)fluoroanthene (3.43), benzo(a)pyrene (5.62), and benzo(g,h,i)perylene (5.44).

## Data Availability

The data presented in this study are available on request from the corresponding author.

## Appendix A

Distance from sampling positions to the industry are detailed in Table A1. Experimental conditions and validation data of PTEs analysis procedure are included in Table A2, Table A3, Table A4 and Table A5 of this Appendix.

**Table A1:** Distance from farm to industry.

Farm Code	Distance (km)					Group
	Zinc	Chemical Fiber	Steel (1)	Steel (2)	Thermal Power Plant	
N1	3.0	5.7	1.9	14	14	Near
N2	5.0	4.1	0.4	12	13	Near
N3	3.4	5.6	1.9	14	14	Near
N4	10	3.7	4.0	6.8	7.2	Near
N5	9.3	0.8	3.4	8.7	10	Near
F1	51	44	45	34	35	Far
N6	7.1	4.0	4.6	13	15	Near
N7	10	4.6	6.6	12	14	Near
N8	5.9	2.8	2.0	12	13	Near
N9	14	5.0	7.2	5.3	7.7	Near
N10	17	9.4	10	0.98	2.3	Near
F2	30	21	24	16	17	Far
F3	27	18	21	13	14	Far
F4	39	32	33	22	23	Far
F5	3.0	5.7	1.9	14	14	Far
F6	5.0	4.1	0.4	12	13	Far

N: Near; F: Far.

**Table A2:** Temperature gradient program used in the acid digestion of soil, forage, and milk.

Time (min)	Power (W)	Temperature (°C)
3	900	95
10	900	160
3	900	185
15	900	185

For each element analyzed by ICP-MS, the LOD and LOQ were calculated as 3 and 10 times the standard deviation (SD) of ten blank samples, respectively.

**Table A3:** Instrumental limit of detection (LOD), limit of quantification (LOQ), and calibration range for inorganic elements quantification by using inductively coupled plasma mass spectrometry.

	LOD	LOQ	Calibration Range
Na mg L^−1^	0.025	0.083	10–200
Mg mg L^−1^	0.001	0.003	10–200
K mg L^−1^	0.132	0.440	10–200
Ca mg L^−1^	0.216	0.720	10–200
Cr µg L^−1^	0.100	0.333	10–5000
Fe µg L^−1^	0.800	2.667	10–5000
Cu µg L^−1^	0.325	1.083	1–50
Zn mg L^−1^	0.002	0.007	0.01–5
As µg L^−1^	0.010	0.033	0.1–50
Se µg L^−1^	0.092	0.305	1–50
Cd µg L^−1^	0.001	0.003	0.1–50
Hg µg L^−1^	0.018	0.060	0.1–10
Pb µg L^−1^	0.017	0.057	0.1–50

Repeatability of extraction procedure from replicates of certified European reference materials was calculated according to the following expression:

$${RSD}_{r}=\frac{\sqrt{\frac{\sum {\left(x_{1}-x_{2}\right)}^{2}}{n-1}}}{\overset{-}{x}}\cdot 100$$

where x_1_ and x_2_ are the measured values for duplicates, n is the number of total measures, and $\overset{-}{x}$ is the average concentration of the element in all the samples.

**Table A4:** Relative standard deviation (RSD) values of soil, ryegrass, and milk powder.

	% Relative Standard Deviation (Repeatability *n* = 3)		
	Soil	Rye Grass	Milk Powder
Na	13.73	1.64	4.42
Mg	2.16	2.26	4.10
K	4.78	1.76	3.53
Ca	2.55	1.18	2.69
Cr	8.75	4.67	23.19
Fe	1.64	5.89	6.13
Cu	1.75	4.63	8.54
Zn	5.02	4.08	3.52
As	2.03	6.77	12.83
Se	4.05	3.31	7.42
Cd	4.67	5.70	5.07
Hg	2.81	2.14	7.76
Pb	2.63	4.65	4.71

In Table A5, validation statistics are included. Note that for Na, Mg, K, and Ca (ERM CD-281 “Rye-grass”) there was no availability of uncertainty values because these inorganic elements were included as additional material information in the ERM CD-281 report. As detailed in this report, the results were obtained from “*semi-quantitative screening analysis using ICP-SFMS (…). The elements were determined using 18 scans over the mass range, resulting in a total measurement time of 300 s. The results are an average of triplicate measurements*”.

**Table A5:** Method validation statistics for ERM-CC141 (loam soil), ERM-CD 281 (rye grass), and ERM BD-151 (skimmed milk powder) analysis by ICP-MS.

	ERM CD-141 “Loam Soil”			ERM CD-281 “Rye Grass”			ERM BD-151 “Skimmed Milk Powder”		
	Cert.	Det. (*n* = 3)	Rec, %	Cert.	Det. (*n* = 3)	Rec, %	Cert.	Det. (*n* = 3)	Rec, %
g kg^−1^									
Na	-	-	-	(4) ^3^	4.07 ± 0.07	102	4.19 ± 0.23	4.39 ± 0.07	105
Mg	-	-	-	(1.6)	1.67 ± 0.03	104	1.26 ± 0.07	1.36 ± 0.03	107
K	-	-	-	(34)	36.3 ± 0.6	107	17 ± 0.8	17.8 ± 0.3	104
Ca	-	-	-	(6.3)	7.2 ± 0.1	114	13.9 ± 0.7	14.1 ± 0.2	101
mg kg^−1^									
Cr	31 ± 4	46 ± 3	149	24.8 ± 1.3	25.4 ± 0.5	102	0.19 ^2^	0.214 ± 0.005	113
Fe	-	-	-	-	-	-	53 ± 4	49.9 ± 0.7	94
Cu	12.4 ± 0.9	12.0 ± 0.1	96	10.2 ± 0.5	10.1 ± 0.2	99	5 ± 0.23	4.52 ± 0.11	90
Zn	50 ± 4	58 ± 1.3	117	30.5 ± 1.1	29.3 ± 0.5	96	44.9 ± 2.3	40.6 ± 0.9	90
As	7.5 ± 1.4	9.6 ± 1.2	128	0.042 ± 0.01	0.046 ± 0.002	109	-	-	-
Se	-	-	-	0.092 ^1^	0.097 ± 0.008	105	0.19 ± 0.04	0.176 ± 0.011	93
Cd	0.25 ± 0.04	0.27 ± 0.01	106	0.120 ± 0.007	0.111 ± 0.004	93	0.106 ± 0.013	0.100 ± 0.003	94
Hg	0.080 ± 0.008	0.087 ± 0.010	108	0.016 ± 0.002	0.018 ± 0.001	112	0.52 ± 0.04	0.475 ± 0.010	91
Pb	32.2 ± 1.4	35.4 ± 1.2	110	1.67 ± 0.11	1.63 ± 0.03	97	0.207 ± 0.014	0.183 ± 0.004	88

^1^ Selenium was added to rye grass ERM before acid digestion. ^2^ Chromium was added to skimmed milk powder ERM before acid digestion. ^3^ Data between brackets indicate approximate values.

PAH quantification was carried out by using GC-MS. A representative chromatogram of a soil sample was included in Figure A1.
